# Dominant contribution of atmospheric nonlinearities to ENSO asymmetry and extreme El Niño events

**DOI:** 10.1038/s41598-024-58803-3

**Published:** 2024-04-07

**Authors:** G. Srinivas, J. Vialard, F. Liu, A. Voldoire, T. Izumo, E. Guilyardi, M. Lengaigne

**Affiliations:** 1 CSIR-National Institute of Oceanography, Dona Paula, Goa 403004 India; 2 LOCEAN-IPSL, Sorbonne Université -CNRS-IRD-MNHN, Paris, France; 3 CIC-FEMD/ILCEC, Key Laboratory of Meteorological Disaster of Ministry of Education (KLME), Nanjing University of Information Science and Technology, Nanjing, China; 4 CNRM, CNRS, Météo‐France, Université de Toulouse, Toulouse, France; 5 IRD, UMR241 (IRD-UPF-ILM-Ifremer), Tahiti, French Polynesia; 6 NCAS-Climate, University of Reading, Reading, UK; 7 MARBEC, CNRS, IFREMER, IRD, University of Montpellier, Sète, France

**Keywords:** El Niño, La Niña, SST, Rainfall, Wind stress, Atmospheric nonlinearities, Asymmetries, Climate sciences, Ocean sciences

## Abstract

Extreme El Niño events have outsized impacts and strongly contribute to the El Niño Southern Oscillation (ENSO) warm/cold phase asymmetries. There is currently no consensus on the respective importance of oceanic and atmospheric nonlinearities for those asymmetries. Here, we use atmospheric and oceanic general circulation models that reproduce ENSO asymmetries well to quantify the atmospheric nonlinearities contribution. The linear and nonlinear components of the wind stress response to Sea Surface Temperature (SST) anomalies are isolated using ensemble atmospheric experiments, and used to force oceanic experiments. The wind stress-SST nonlinearity is dominated by the deep atmospheric convective response to SST. This wind-stress nonlinearity contributes to ~ 40% of the peak amplitude of extreme El Niño events and ~ 55% of the prolonged eastern Pacific warming they generate until the following summer. This large contribution arises because nonlinearities consistently drive equatorial westerly anomalies, while the larger linear component is made less efficient by easterly anomalies in the western Pacific during fall and winter. Overall, wind-stress nonlinearities fully account for the eastern Pacific positive ENSO skewness. Our findings underscore the pivotal role of atmospheric nonlinearities in shaping extreme El Niño events and, more generally, ENSO asymmetry.

## Introduction

Extreme El Niño events, like those during 1982–1983, 1997–1998 and 2015–2016, considerably impact the tropical Pacific’s deep atmospheric convection, influencing regional hydro-climatic conditions and extreme weather events, resulting in outsized societal impacts^1,2^. These events result in a distinct, eastward-shifted teleconnection pattern over Northern America, increasing the likelihood of heat waves over northern USA and Canada^3^ and of wet years over California^3–5^. These wetter conditions relate to a prolonged eastern Pacific warming until the subsequent summer during extreme events^6^, while warm anomalies recede by spring during moderate events. The strong and distinctive impacts of extreme El Niño events make their specific dynamics a prominent area of research.

Extreme El Niño events strongly enhance the overall El Niño/Southern Oscillation (ENSO) asymmetry^7,8^, with their large amplitude in the eastern Pacific indeed intensifying and shifting the El Niño signals eastward relative to those of La Niña^9^. The ENSO phase transition also displays asymmetry^10^, often transitioning from extreme El Niño to La Niña, while La Niña events can persist for multiple years, as exemplified by *triple-dip La Niña*^11^ from mid-2020 to early 2023. ENSO amplitude, spatial pattern and duration asymmetries are closely tied to its diversity^12^. El Niño events are commonly partitioned into two categories, characterized by sea surface temperature (SST) anomalies peaking either in the central (dateline, Modoki or Central Pacific type) or eastern (Eastern Pacific type) Pacific. In contrast, the La Niña events display less diversity. Nonlinear processes play a pivotal role in shaping ENSO asymmetry^9,13^ and extreme El Niño events^14,15^ but there is currently no consensus regarding the primary contributors to ENSO asymmetries and extreme El Niño events, whether atmospheric nonlinearities, oceanic nonlinearities or both.

Deep atmospheric convection response to SST is the primary source of tropical atmospheric nonlinearity, with convection generally occurring above a 27.5 °C threshold^16,17^. Climatological SST rises westward across the equatorial Pacific, crossing this threshold near the dateline. Consequently, positive SST anomalies enhance convection east of the dateline, while negative anomalies suppress it westward, causing the eastward shift of El Niño’s wet anomalies relative to La Niña dry anomalies^18^. Convective nonlinearities contribute to ~ 40% of eastern Pacific rainfall anomalies during extreme El Niño events^19^, leading to a stronger and eastward-shifted wind-stress response during El Niño. This response has been proposed to strongly contribute to ENSO asymmetry^20–23^ and the genesis of extreme El Niño events^15,24^. The enhanced wind-stress response during El Niño also operates at sub-seasonal timescales. Short-lived reversals of the trade winds in the western and central Pacific (Westerly Wind Events) act as a stochastic forcing of ENSO^25^ and are more frequent and eastward-shifted during El Niño events^26,27^, contributing to atmospheric nonlinearity. This ENSO-modulated stochastic forcing, termed *multiplicative noise*, also contributes to the ENSO amplitude asymmetry^28,29^.

Various oceanic sources of nonlinearity have also been proposed to contribute to ENSO asymmetries. One is the thermocline feedback nonlinearity linked to potentially larger thermocline depth and subsurface anomalies during El Niño than during La Niña, for which the surface bounds the thermocline upward movement. Integrating this nonlinearity into simple ocean models yields larger El Niño than La Niña events^14,30^. Oceanic advection is also a source of nonlinearities. Tropical Instability Waves (TIWs) form on the northern edge of the cold tongue, and transport heat towards the equator. ENSO influences TIWs activity, inducing weak anomalous cooling during El Niño and anomalous strong warming during La Niña, thereby contributing to ENSO asymmetry^31,32^. At lower frequencies, nonlinear advection terms (advection of SST anomalies by current anomalies, referred to as nonlinear dynamical heating or NDH), tend to warm during both El Niño and La Niña. NDH is thought to contribute to positive ENSO skewness^33^. Additional oceanic mechanisms include the asymmetrical response of oceanic Kelvin and Rossby waves to wind-stress forcing^34^ and bio-physical feedback^35^. Multiple oceanic and atmospheric mechanisms have thus been proposed to explain ENSO asymmetry and the emergence of extreme El Niño events. Nevertheless, a consensus on their relative importance remains elusive. A recent study by Geng et al.^13^ suggests that atmospheric nonlinearities play a dominant role in ENSO amplitude asymmetry, while oceanic nonlinearities contribute less. However, these results relied on an idealized model and this study did not specifically address extreme El Niño events.

Our study aims to quantify the contribution of atmospheric nonlinearities to ENSO asymmetries and extreme El Niño events. To that end, we use forced experiments with state-of-the-art oceanic and atmospheric general circulation models that accurately reproduce the ENSO signals and asymmetries. In “Atmospheric nonlinear response to SST” section, dedicated ensemble atmospheric experiments allow isolating the nonlinear component of the wind-stress response to SST, underscoring the role of deep convection in this nonlinearity. In “Large contribution of atmospheric nonlinearities to ENSO asymmetry and extreme El Niño events” section, we separately apply the linear and nonlinear components of the wind-stress anomalies to oceanic experiments, emphasizing the dominant role of wind-stress nonlinearities for eastern Pacific ENSO asymmetries, particularly during the peak and decay phases of extreme El Niño events. Finally, “Discussion” section provides a summary and discussion of our results.

## Atmospheric nonlinear response to SST

In this section, we analyse ensemble experiments with the atmospheric component of the CNRM-CM6.1 climate model, known as Arpege^36^. We first demonstrate that these experiments reproduce the observed wind-stress and rainfall ENSO asymmetries accurately. We then estimate the linear and nonlinear wind-stress response to SST from two ensemble experiments forced by observed SST anomalies and their opposite (see “Methods” section for details). We finally demonstrate the central role of the nonlinear relation between tropical rainfall and SST in establishing the nonlinear wind-stress response to SST.

**Figure 1:**
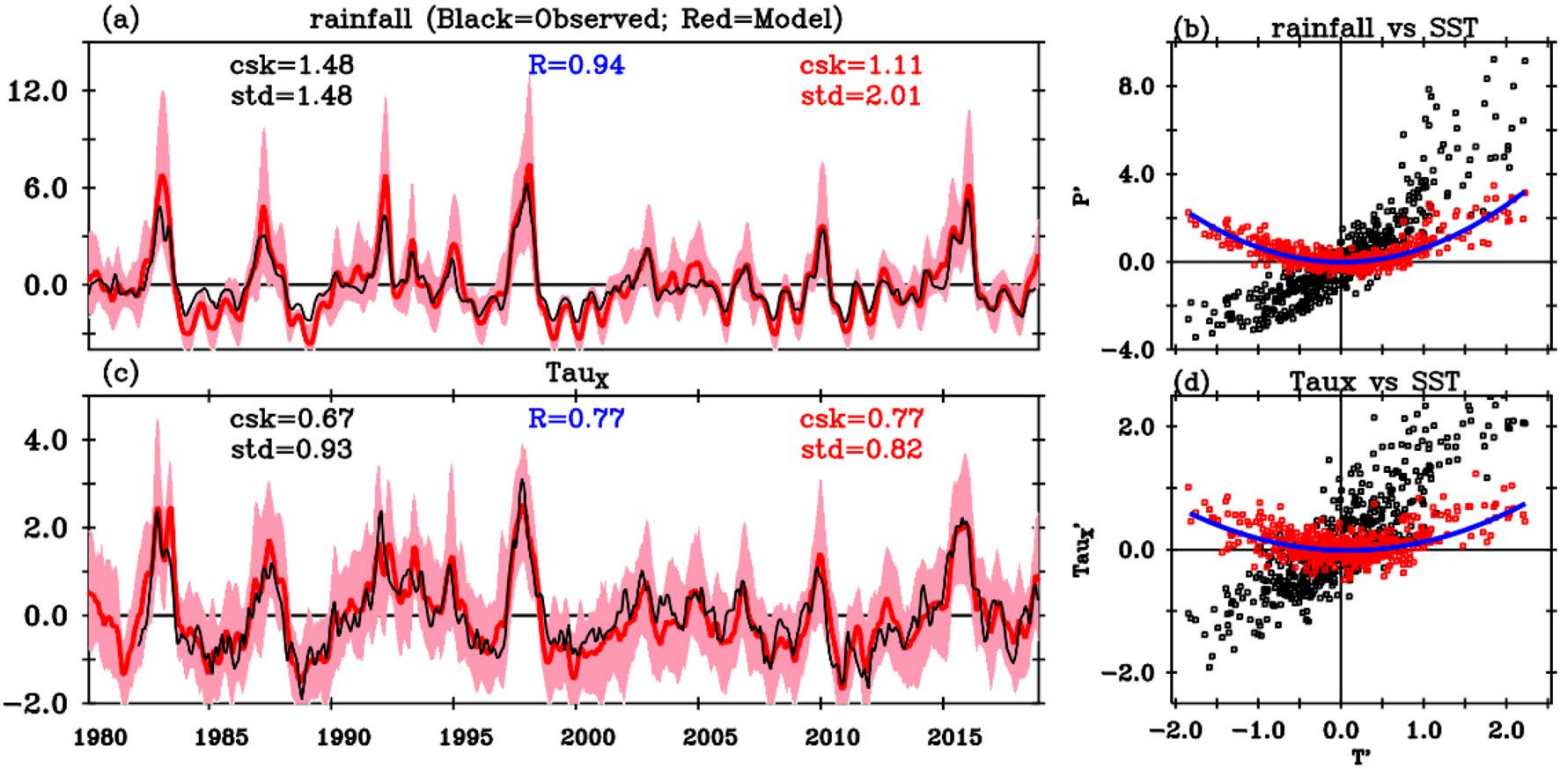
(**a**) Time series of interannual anomalies of precipitation (mm/day) averaged over the Niño3 + 4 region (160° E–90° W, 5° N–5° S) for the control AGCM simulation (red curve) and observations (black curve). The red shading represents the ensemble spread of the control AGCM simulation (10th and 90th percentiles). (**b**) Scatter diagram of simulated anomalous rainfall response to SST anomalies over the Niño3 + 4 region (black dots) with its nonlinear contribution (red dots, see “Methods” section for details). The blue curve is a quadratic fit of the atmospheric nonlinear response to SST anomalies. (**c,d**) Same as (**a,b**) but for zonal wind-stress anomalies (10^–2^ Nm^−2^). The standard deviation (std) and coefficient of skewness (csk) are indicated (AGCM ensemble mean in red, observations in black), as well as the correlation (R) between the AGCM ensemble mean and observed time series.

**Figure 2:**
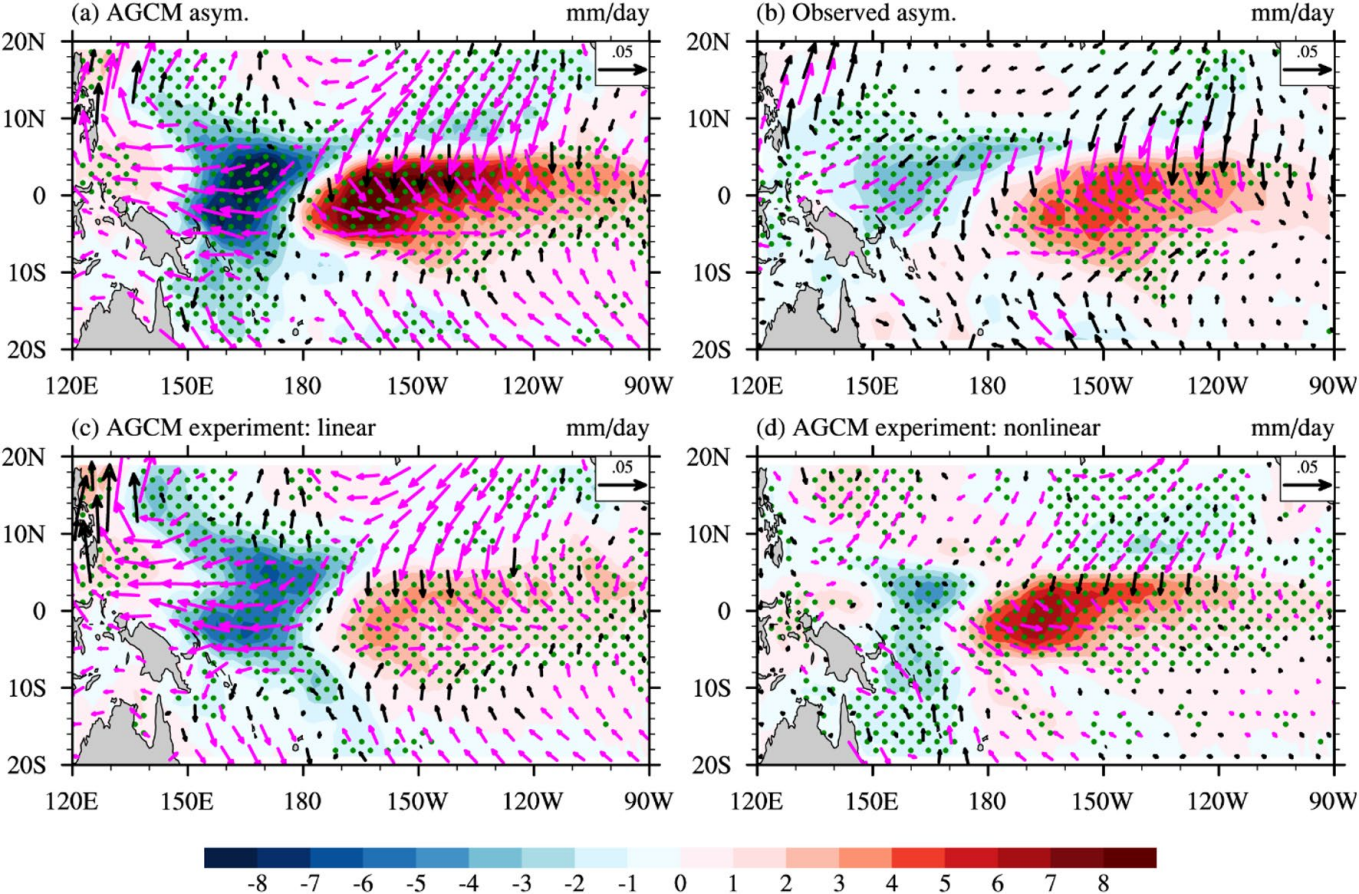
(**a**) December–February (DJF) average ENSO asymmetries (obtained as the half sum of extreme El Niño and strong La Niña composites, see “Methods” section for details) the control GCM simulation rainfall (shaded, mm/day) and wind stress (vectors, Nm^−2^) and contributions from the (**c**) linear and (**d**) nonlinear response to SST. See text and Supplementary Fig. S3 for details of the decomposition into linear and nonlinear responses to SST. (**b**) It is the same as (**a**) but for observations. The green stippling (magenta vectors) indicates the SST (zonal wind stress) asymmetry that is different from zero at the 95% confidence level based on a t-test.

Figure 1a,c shows the time series of observed and simulated interannual rainfall and wind-stress anomalies over the Niño3 + 4 region (160° E–90° W, 5° N–5° S). This region, similar to that used in Jin et al.^37^, encompasses the wind-stress and rainfall strong El Niño response, which peaks in Niño3, and the La Niña response, which peaks in Niño4. The modelled evolution closely aligns with observations, which typically fall within their ensemble spread (also see suppl. Figs. S1, S2, which indicate a realistic wind stress-SST sensitivity of 0.016 Nm^−2^ K^−1^ against 0.013 to 0.016 in observational products). Figure 2a,b illustrates observed and simulated ENSO rainfall and wind-stress asymmetries, computed as the half sum of extreme El Niño and strong La Niña composites (see methods for definition; a similar pattern emerges when considering all events, not shown). Observed east–west rainfall and zonal wind-stress dipoles indicate that the El Niño wet and westerly anomalies are stronger and eastward-shifted compared to the La Niña dry and easterly anomalies (Fig. 2b). The model reproduces these observed wind-stress and rainfall asymmetry patterns exhibiting (pattern correlations of 0.75 and 0.77, respectively) although with an overestimated model rainfall asymmetry amplitude (Fig. 2a). The above analyses collectively indicate that control ensemble atmospheric experiments effectively capture the surface atmospheric ENSO variations and asymmetries.

Figure 1b,d displays scatterplots of total (black dots) and nonlinear (red dots) rainfall and wind-stress versus SST anomalies over the Niño3 + 4 region. The nonlinear component of the atmospheric response to SST anomalies is obtained from the half-sum of ensemble means of experiments forced by observed SST anomalies and their opposite (for more details, refer to the “Methods” section). The linear component is obtained from their difference. While theoretically, this approach should separate odd and even terms in the Taylor expansion, in practice, the 3rd and higher-order terms are negligible (not shown). Consequently, we will simply refer to the “linear” and “nonlinear” parts of the atmospheric response to SST. Note that in such averaged ensemble analysis, *multiplicative noise forcing* associated with Westerly Wind Events appears as a smooth seasonal rather than a high-frequency nonlinear component.

The relationship between rainfall and SST anomalies (Fig. 1b) demonstrates a pronounced nonlinearity: positive SST anomalies are more effective in triggering the wet anomalies, resulting in rainfall anomalies that are nearly twice as large for a 2 °C warming than for a 2 °C cooling. This nonlinearity is associated with the convexity of the precipitation-SST relationship (see Supplementary Fig. S3), and can be attributed to the anomalous convergence of moisture anomalies and the convergence feedback^19,38^. Consequently, the nonlinear response of rainfall to SST exhibits a primarily quadratic shape (red dots in Fig. 1b), leading to amplified wet anomalies during El Niño and reduced dry anomalies during La Niña. Similarly, zonal wind-stress nonlinearities also display a quadratic dependence on SST (Fig. 1d), which is similar to that in observations (Supplementary Fig. S2). Zonal wind-stress indirectly depends on SST through the following loop: (i) it depends on surface wind through a nonlinear bulk formula; (ii) surface wind depends on tropospheric heating through Gill-type dynamics; and (iii) rainfall depends on SST through the relation shown in Fig. 1b. Relations (i) and (ii) are almost linear (see Suppl. Fig. S4b,c). The nonlinearity in the wind-stress/SST dependence hence mainly arises from that in the rainfall-SST relationship (Fig. S4d). In practical terms, wind-stress exhibits a quadratic dependence on SST similar to that of rainfall, amplifying westerly wind-stress anomalies during El Niño and reducing easterly wind-stress anomalies during La Niña (Fig. 1d).

Figure 2c,d decomposes the ENSO peak asymmetry displayed in Fig. 2a into contributions from the linear and nonlinear response to SST. For rainfall, the linear contribution (Fig. 2c) is primarily associated with a zonal shift in rainfall pattern. This shift is simply a linear response to the westward-shifted SST pattern during the La Niña peak, and the eastward-shifted pattern during El Niño. This zonal shift does not contribute strongly to the zonally-averaged equatorial rainfall asymmetry (only 5% of the 1.45 mm day^−1^ asymmetry over Niño3 + 4). In contrast, atmospheric nonlinearities predominantly lead to a positive rainfall anomaly (Fig. 2d, the negative anomalies around 160° E being weaker and over a more limited region than positive anomalies further east), attributed to the positive nonlinear rainfall contribution during both El Niño and La Niña (Fig. 1b). Consequently, atmospheric nonlinearities dominate the zonally-averaged rainfall asymmetry (95% over the Niño3 + 4 region). Given that the main nonlinearity in the wind-stress response arises from the rainfall/SST relation, wind-stress behaves similarly to rainfall. The linear response to SST mostly involves a zonal shift in the wind-stress pattern (0% of the total 0.003 Nm^−2^ total zonally-averaged wind-stress asymmetry), while the nonlinear response governs the asymmetry in zonally-averaged zonal wind-stress (100%). Overall, this analysis underscores that atmospheric nonlinearities induce a zonal-mean equatorial westerly anomaly during the El Niño and La Niña peaks, while the larger linear contribution is mostly associated with a zonal shift that hardly contributes to the zonal-mean anomaly during and shortly before the ENSO peak season.

## Large contribution of atmospheric nonlinearities to ENSO asymmetry and extreme El Niño events

The zonally-averaged westerly wind-stress anomaly resulting from atmospheric nonlinearities discussed in the previous section should amplify El Niño and damp La Niña events. However, previous studies have proposed that the eastward shift of the linear wind-stress response during El Niño events can also contribute to ENSO asymmetries^20^. To quantify these two effects, this section examines ocean general circulation model (OGCM) experiments forced by the linear (characterized by a zonal shift between El Niño and La Niña peaks) and nonlinear (involving zonally-averaged westerlies) components of the wind-stress response to SST (see “Methods” section).

**Figure 3:**
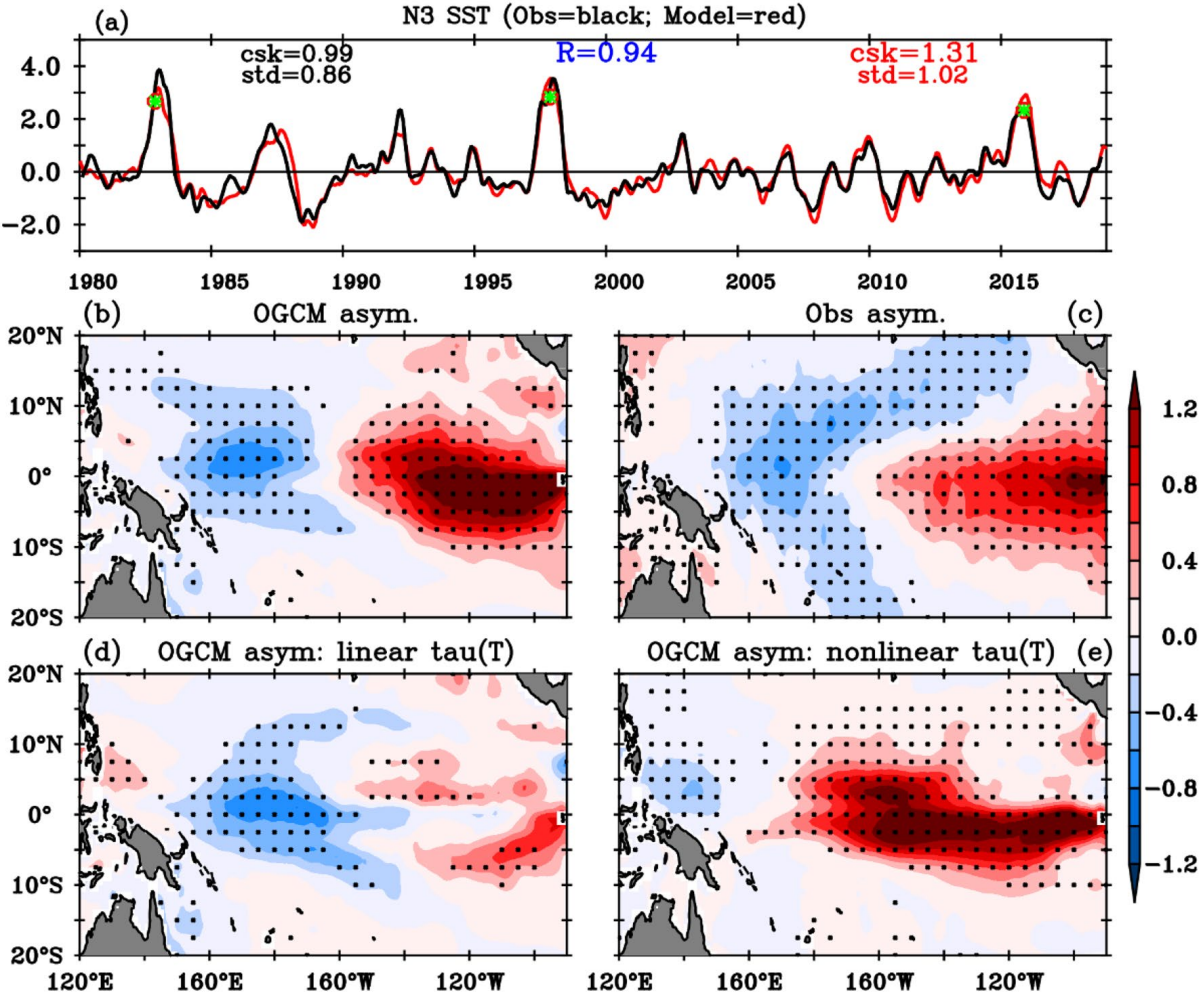
(**a**) Time series of the average Niño3 (5° N–5° S, 150° W–90° W) SST anomalies for the control OGCM simulation (red curve) and observations (black curve). (**b**) DJF average ENSO SST asymmetries (shaded, ℃, obtained as the half sum of extreme El Niño and strong La Niña composites, see text for details) in the OGCM control simulation, and contributions from the (**d**) linear and (**e**) nonlinear wind stress response to SST. See the text for details on the ocean model experiments that yield (**d,e**). Panel (**c**) is the same as (**b**) but for observations. The statistical quantities on panel (**a**) show standard deviation (std), coefficient of skewness (csk) and correlation coefficient (R) values. Stippling on (**b–e**) indicates asymmetry that is different from zero at the 95% confidence level based on a t-test.

To begin, we assess the control ocean experiment’s ability to reproduce ENSO asymmetries and strong events. The model agrees very well with observed SST anomalies time evolution in Niño3 (Fig. 3a), with correlations above 0.94 and comparable amplitude and skewness. The simulation accurately reproduces observed equatorial SST anomalies solely by prescribing wind-stress, and modelling heat flux interannual anomalies as a simple linear damping (see “Methods” section for details). Figure 3bc specifically focuses on oceanic asymmetries between El Niño and La Niña. As previously discussed for rainfall and wind-stress, the east–west dipole indicates a larger SST response during El Niño in the east and during La Niña in the west. The model faithfully replicates SST ENSO asymmetries very well, with a pattern correlation of 0.8.

In the following, we analyse two sensitivity experiments (see “Methods” section for details). The “linear τ(SST)” experiment is similar to the control experiment above, but with the nonlinear wind-stress response to SST diagnosed from the AGCM experiments ${\overrightarrow{\tau }}_{nl}^{\prime}$ subtracted from the wind stress forcing. The JRA55-do wind stress anomalies that force the control OGCM are very similar to those provided by our AGCM experiments (Fig. 1, Supplementary Figs. S1, S2), justifying our approach (more on this in the “Discussion” section). The “nonlinear τ(SST)” OGCM experiment aims at isolating the oceanic response to the nonlinear component of wind stress anomalies, and is forced by climatological wind-stress from the control oceanic experiment plus the nonlinear wind-stress anomalies ${\overrightarrow{\tau }}_{nl}^{\prime}$. Wind stress anomalies from the linear and nonlinear τ(SST) experiments by construction add up to those of the OGCM control experiment. Figure 3d,e allows comparing the SST asymmetry in the linear and nonlinear τ(SST) experiments to that in the control experiment (Fig. 3b). The linear τ(SST) experiment has a strongly reduced SST positive skewness in the eastern Pacific, with milder impact on the SST asymmetry in the western Pacific. In contrast, the nonlinear τ(SST) experiment has a large skewness in the eastern half of the Pacific, surpassing that of the control experiment in the central Pacific (we will explain this below). This implies that atmospheric nonlinearities are primarily responsible for the eastern Pacific SST positive skewness, while the negative skewness in the western Pacific is attributable to the zonal shift in the linear wind-stress response and/or oceanic nonlinearities. Overall, this analysis underscores the dominant contribution of wind-stress/SST nonlinearities to the larger El Niño than La Niña amplitude in the eastern Pacific.

**Figure 4:**
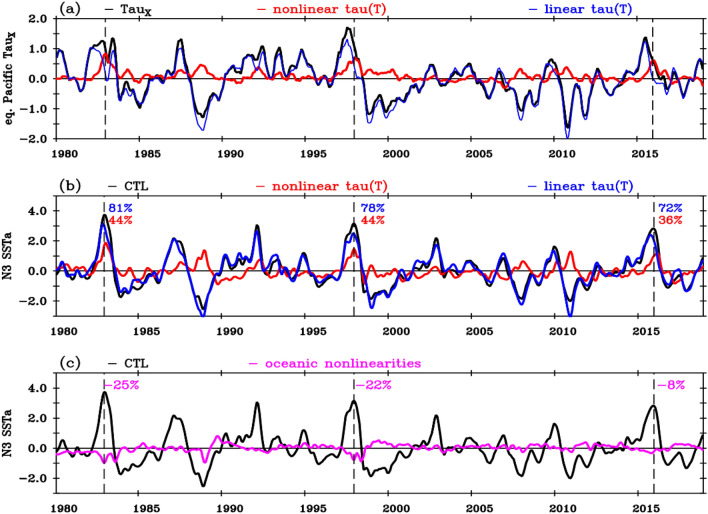
Interannual time series of (**a**) equatorial Pacific (5° N–5° S, 120° E–80° W) averaged zonal wind stress anomalies (10^–2^ Nm^−2^) with contributions from the linear (blue curve) and nonlinear (red curve) components of the response to SST. Niño3 SST anomalies (°C) from (**b**) the control OGCM simulation (black), and contributions from the linear (blue, linear τ(SST) experiment) and nonlinear (red, nonlinear τ(SST) experiment) wind stress response to SST, (**c**) the control OGCM (black) and the contribution from oceanic nonlinearities (magenta curve, obtained as the control minus the sum of the linear and nonlinear τ(SST) experiments). The vertical dashed lines mark the peak of the three extreme El Niño events in December 1982, 1997 and 2015. The numbers indicate the percentage of the contributions of the linear (blue), nonlinear (red) wind stress components and of oceanic nonlinearities (magenta) to the total November–January Niño3 SST anomalies during these three events (see Supplementary Table S1 for details). The average contribution over the three extreme El Niño events is 77% for linear τ(SST), 41% for nonlinear τ(SST), and − 18% for oceanic nonlinearities.

In this paragraph, we delve into the specific contribution of atmospheric nonlinearities during extreme El Niño events. Figure 4a provides a time series of zonally-averaged equatorial Pacific zonal wind-stress anomalies, which are further broken down into contributions from the linear (blue) and nonlinear (red) components of the wind-stress response to SST. As anticipated from Fig. 2d, nonlinearities predominantly lead to zonally-averaged westerly wind-stress anomalies during extreme El Niño events (see zooms in Supplementary Fig. S5). The linear wind-stress component dominates the total anomalies during most of the ENSO lifecycle, except around the peak phase of extreme El Niño events (1982–1983, 1997–1998 and 2015–2016, marked by dashed purple lines) and to a lesser extent during strong La Niña events. Given that atmospheric nonlinearities have the most substantial influence on SST asymmetry in the east, we now focus on the Niño3 box. Figure 4b shows the SST response to the wind-stress anomalies linear (blue) and nonlinear (red) components in our ocean experiments. The SST response echoes the behaviour of the wind-stress forcing, with the linear part prevailing most of the time. However, atmospheric nonlinearities strongly contribute to the eastern Pacific warming during and after the end of the three extreme El Niño events (Fig. 4b, also see Supplementary Fig. S5 zooms). During the peak phase, wind-stress nonlinearities consistently account for about 45% of the total SST anomalies (Fig. 4b, Supplementary Table S1). Furthermore, extreme El Niño events are followed by a prolonged eastern Pacific SST warming that lasts until the following summer^39–41^. Wind stress nonlinearities contribute to ~ 55% of this prolonged warming (Fig. 4b, Table S1). Overall, the wind stress-SST nonlinearity contributes to ~ 40% of the amplitude of SST anomalies during and ~ 55% shortly after the peak of extreme El Niño events.

Wind stress anomalies of the linear and nonlinear τ(SST) experiments add up to those of the control experiment. Any difference between the sum of SST anomalies in the linear and nonlinear τ(SST) experiments and the control is thus attributable to oceanic nonlinearities (magenta curve on Fig. 4c). Oceanic nonlinearities act to weakly dampen the peak of extreme El Niño events (by about 15%, Fig. 4c, Supplementary Table S1). This confirms the primary role of atmospheric nonlinearities in the genesis of extreme El Niño events, and contrasts with studies that underline that oceanic nonlinearities contribute to the observed ENSO asymmetry^31–33^: we will come back to this in the “Discussion” section.

**Figure 5:**
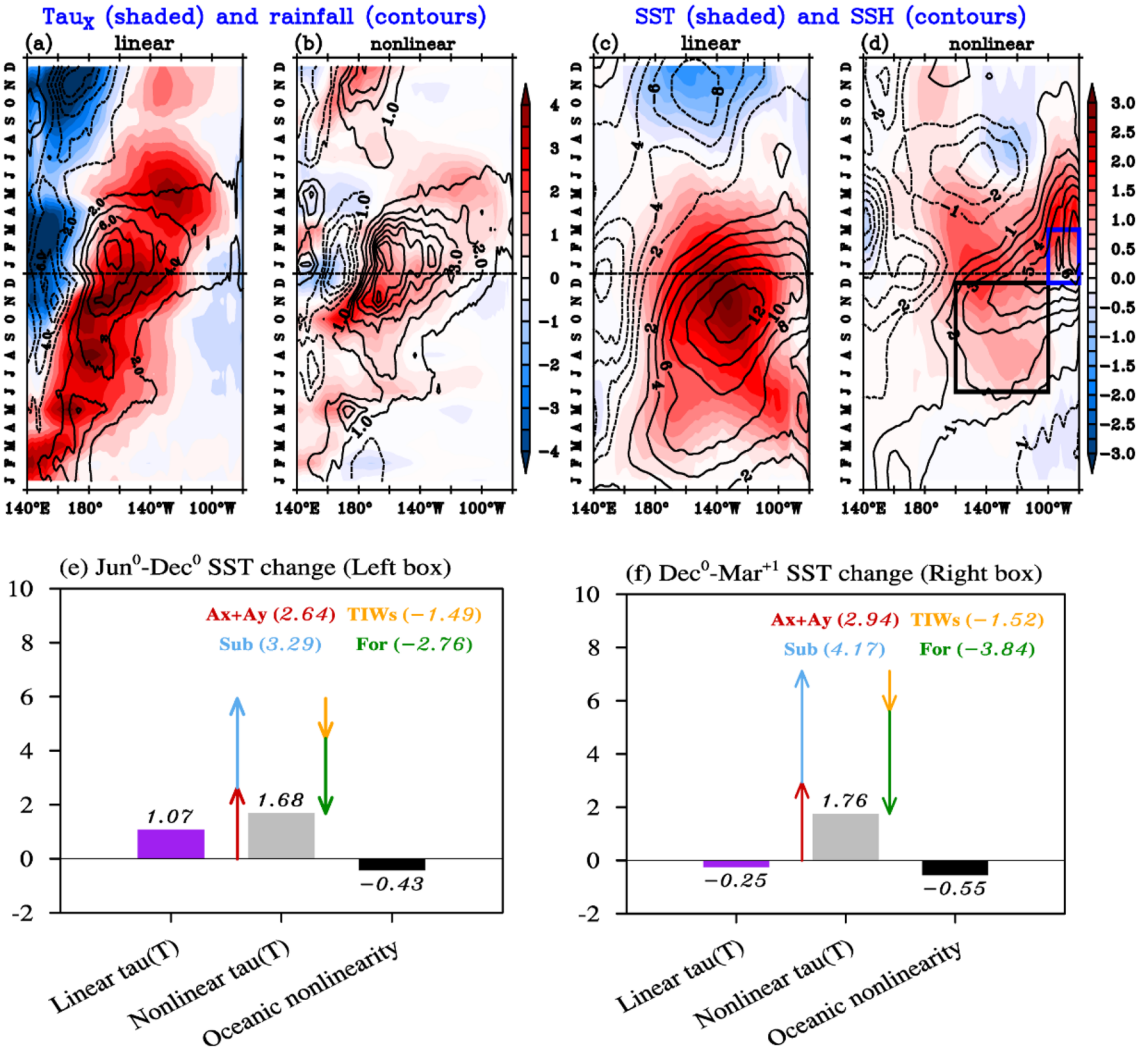
Composite life-cycle of anomalies attributable to the linear and nonlinear wind stress response to SST anomalies during extreme El Niño events. Longitude-time section of equatorial (5° N–5° S) average (**a**) linear and (**b**) nonlinear zonal wind stress (shaded, 10^–2^ Nm^−2^) and rainfall (contours, mm/day) response to SST anomalies; and SST (shaded, ℃) and SSH (contours, cm) anomalous responses to the (**c**) linear (linear τ(SST) experiment) and (**d**) nonlinear (nonlinear τ(SST) experiment) components of the wind stress. The left (black, 100 to 160° W, 5° N to 5° S) and right (blue, 90 to 100° W, 5° N to 5° S) boxes on panel (**d**) respectively indicate the regions and periods over which the spatially averaged and temporally integrated heat budgets of panels (**e,f**) are performed. Those budgets indicate the total contribution of the linear (purple) and nonlinear (grey) wind stress and the oceanic nonlinearities (black) to the SST change (i.e. SST anomalies at the end minus those at the beginning of the period). The contribution of the nonlinear wind stress to the SST change is further broken into lateral advection (red), subsurface processes (blue), air-sea fluxes (green) and Tropical Instability Waves (yellow). Panel (**e**) focuses on the central and eastern Pacific warming in the 6 months that precede the El Niño peak (i.e. 1st June 1997 to 1st December 1997, see black box on panels (**d,f**) on the prolonged eastern Pacific warming during the 4 months that follow the El Niño peak (i.e. 1st December 1997 to 1st April 1997, see black box on panel **d**). Panels (**a,b**) are obtained from the ensemble AGCM experiments and (**c–f**) from the OGCM experiments described in the text.

We now investigate a composite of the three extreme El Niño events to understand the oceanic mechanisms at play, with a specific focus on the effect of the nonlinear wind-stress response to SST. Figure 5a–d displays zonal-time sections of the equatorial atmospheric linear and nonlinear anomalies and their oceanic response for the extreme El Niño composite. ENSO linear dynamics (Fig. 5a,c) are well-established and only briefly recapped here. During the onset phase, the central Pacific warming (Fig. 5c) initiates enhanced rainfall and westerlies in the western Pacific (Fig. 5a), triggering downwelling Kelvin waves (Fig. 5c). This, in turn, sets off zonal advective and thermocline feedbacks that further increase the central and eastern Pacific warming^37^ (Fig. 5c). However, from boreal fall, the linear dynamics become less effective in amplifying the warming for two reasons. The first is the well-known delayed negative feedback associated with equatorial wave reflections at meridional boundaries and the Recharge Oscillator dynamics of ENSO^42,43^. Yet Fig. 5 also points to the role of easterly anomalies in the western Pacific during large El Niño events^44^. As discussed in “Atmospheric nonlinear response to SST” section, the linear response to SST during the peak phase of extreme El Niño events is associated with enhanced rainfall in the central Pacific (tropospheric heat source), but a suppression of western Pacific rainfall (a heat sink). This western Pacific heat sink leads to western Pacific easterlies during the peak phase (Fig. 5a). Consequently, the east Pacific oceanic response to the linear wind-stress westerly component is partially compensated by the opposing easterly anomaly further west, with a ~ 2-month delay attributable to the travel time of first baroclinic mode Kelvin waves from the western to the eastern equatorial Pacific, making it less efficient.

We will now discuss the impact of atmospheric nonlinearities (Fig. 5b,d) during the extreme El Niño peak phase. The nonlinear rainfall and wind-stress anomalies become most prominent during the peak and decay phases (Fig. 5b) when the large SST anomalies in the eastern Pacific exceed the convective threshold. As discussed earlier, these nonlinear contributions are associated with zonally-averaged rainy and westerly anomalies. The sea level response to this nonlinear westerly anomaly becomes as large as the linear part during the ENSO peak and larger after the peak, for two reasons. First, unlike the ~ 10-month-old linear component, the younger response to the nonlinear atmospheric forcing is not yet affected by the delayed negative feedback associated with oceanic dynamics. Second, unlike the linear part that displays a dipole in zonal wind-stress, the nonlinear part is very predominantly westerly (Fig. 5b). Note that the nonlinear rainfall and wind stress anomalies also involve a zonal dipole (Fig. 5a,b), but with a much weaker western Pacific dry, easterly pole, leading less compensation than for the linear part (Fig. 5ab). Consequently, the zonally-averaged nonlinear wind-stress component (Fig. 5b) surpasses the linear one during and after the peak of extreme El Niño events (Fig. 4a, Supplementary Fig. S5 zooms: the October–March zonally-averaged nonlinear component is 0.006 Nm^−2^ against 0.002 Nm^−2^ for the linear one). This leads to a downwelling Kelvin wave response to the nonlinear wind-stress components peaking at the end of the year (contours on Fig. 5d), while the linear component (contours on Fig. 5c) is already decreasing due to delayed oceanic negative feedback and the presence of easterlies in the western Pacific. Figure 5e shows the central and eastern Pacific mixed layer heat budget (black box in Fig. 5d) during June-December (see “Methods” section for details), shedding light on how this ocean dynamical response translates into warming. This analysis indicates that the nonlinear atmospheric processes have contributed to 50% more June to December eastern Pacific warming than the linear ones in the central-eastern Pacific (with a weak negative contribution from oceanic nonlinearities, as mentioned earlier). Note that this analysis is focussed on the period over which the nonlinear wind stress response to SST dominates the budget, and hence gives a larger contribution of nonlinear atmospheric processes than Fig. 4 and Table S1, which are better estimates of the overall contribution to extreme El Niño peak anomalies. The response to atmospheric nonlinearities can further be decomposed into various processes, and display a very similar balance of mechanisms to that described in Vialard et al.^45^ for the total SST anomalies evolution during the extreme 1997–1998 El Niño. The nonlinear westerly anomalies induce warming through subsurface oceanic processes (Fig. 5e, blue arrow) via the thermocline feedback, and through lateral advection (Fig. 5e, red arrow) via the zonal advective feedback. Those two warming effects are partly counterbalanced by the thermal damping associated with air-sea fluxes (Fig. 5e, green arrow) and by the decreased heat flux convergence to the cold tongue in response to the Tropical Instability Waves (TIWs) suppression (Fig. 5f, yellow arrow).

Let us now discuss the nonlinear contribution to the prolonged eastern Pacific warming, which extends until June-July following the peak of extreme El Niño events. Our analysis indicates that atmospheric nonlinearities are the sole contributors to this warming’s initial phase (Fig. 5c,d). This is confirmed by the December–March oceanic mixed layer heat budget (blue box on Fig. 5d) in the eastern Pacific (Fig. 5f): the response to nonlinear wind stress strongly warms the ocean, while the linear wind stress induces a modest cooling, as well as oceanic nonlinearities. As previously mentioned, this is due to the reduced linear wind stress efficiency in association with the delayed negative oceanic feedback and easterly anomalies in the western Pacific. In contrast, the persistent eastern Pacific warming associated with atmospheric nonlinearities triggers rain and westerly anomalies in the central-eastern Pacific (Fig. 5b,d) that force a downwelling response until summer in the far eastern Pacific (Fig. 5d). Figure 5f indicates that the combination of processes associated with this warming induced by atmospheric nonlinearities closely resembles the one for the peak phase, involving warming through the thermocline and advective feedbacks and cooling through air-sea fluxes thermal damping and the suppression of TIWs.

In this section, we demonstrated that the westerly wind-stress anomalies associated with the wind-stress/SST nonlinearity play a crucial role in generating the positively skewed SST anomaly in the eastern Pacific. This effect is largest during extreme El Niño events when it contributes to ~ 40% of the peak Niño3 and ~ 55% of the eastern Pacific prolonged warming amplitude. Although the nonlinear component of the wind-stress is in general relatively modest (Fig. 5b), it is consistently westerly and is not offset by opposing anomalies in the western Pacific, as happens for the linear wind-stress response to SST during and after extreme El Niño events peaks. Consequently, this nonlinear wind-stress response deepens the eastern Pacific thermocline from boreal winter to the subsequent summer more effectively, explaining its significant contribution to the dynamics of extreme El Niño events.

## Discussion

### Summary

There is currently no scientific consensus on the relative weights of atmospheric and oceanic nonlinear processes contributing to ENSO asymmetries and the emergence of extreme El Niño events. Here, we emphasize the dominant role of the wind stress-SST nonlinearity induced by the stepwise deep atmospheric convection response to SST.

We evaluate the linear and nonlinear components of the atmospheric (and in particular wind-stress) response to SST by conducting ensemble experiments with the atmospheric component of the CNRM-CM6 climate model, which accurately reproduces ENSO overall asymmetry and the atmospheric signatures of extreme El Niño events. The nonlinear nature of the convective response to SST anomalies leads to a quadratic nonlinearity, characterized by positive rainfall and westerly wind stress anomalies during both El Niño and La Niña, with the most pronounced signature during extreme El Niño events.

We then use oceanic general circulation model experiments with the NEMO v3.6 model^46^. While the control experiment successfully reproduces observed SST skewness and extreme El Niño amplitude in the eastern Pacific, the experiment forced by the linear component of the wind stress has no amplitude asymmetry in this region. This linear wind stress component underestimates both the extreme El Niño peak amplitude and the prolonged eastern Pacific warming until the subsequent summer. The wind stress nonlinearity contributes to ~ 40% of the peak (NDJ) and ~ 55% of the prolonged (FMA) Niño3 SST anomalies during and after extreme El Niño events, due to two main factors. First, the wind stress nonlinear component only becomes strong during boreal fall, when the SST anomaly is large, and is, therefore, less affected by the delayed oceanic negative feedback than its linear counterpart, which has been established since spring. Second, the linear component of the atmospheric response is associated with a large eastward shift of deep atmospheric convection during the peak of extreme El Niño events. This yields an anomalous tropospheric heat sink and western Pacific easterlies in the linear atmospheric response, which makes its contribution to the zonally-averaged equatorial wind stress anomalies weaker than the consistently westerly nonlinear atmospheric response. As a result, the eastern Pacific downwelling Kelvin wave and positive SST response to the nonlinear wind stress become comparable to those of its linear counterpart during the ENSO peak and until the following summer. In conclusion, our study underscores the paramount role of wind-stress nonlinearities in shaping ENSO asymmetries and driving extreme El Niño events.

### Discussion

Our results align with previous research emphasizing the strong influence of atmospheric nonlinearities on ENSO asymmetry and extreme El Niño events^13,15,20–23^. This includes the recognition of the role of multiplicative noise forcing^28,29^, although it appears as smooth seasonal forcing in our study due to ensemble averaging. Notably, our conclusions diverge from those of Kang and Kug^20^, who argued for greater efficiency of eastward shifted wind-stress anomalies during El Niño events. Our study indicates that this eastward shift is balanced out by the emergence of opposite easterly anomalies in the western Pacific during extreme El Niño events.

Our results also underline the prominent role of nonlinear atmospheric processes in the observed eastern Pacific persistent warming after extreme El Niño events. Previous studies^39–41^ propose that this atypical persistent warming is linked to the suppression of eastern equatorial Pacific easterlies, in response to an equatorward shift of the Intertropical Convergence Zone, suppressing equatorial upwelling and warming the surface ocean as a result. Our results consistently indicate that the nonlinear wind stress component weakens central and eastern Pacific easterlies, contributing to anomalous warming through subdued upwelling and mixing. We additionally highlight the role played by anomalous advection in driving this prolonged warming.

The validity of our results is contingent upon the CNRM-CM6 atmospheric component providing a reasonable estimate of the wind stress nonlinearity. The excellent comparison between the ensemble average equatorial Pacific wind stress variations (including their nonlinear component) and observed estimates (Fig. 1, Suppl. Figs. S1, S2), as well as their ENSO warm-cold phases asymmetry (Fig. 2ab), suggests that this is the case. The AGCM wind stress feedback may however be overestimated by 10–20% and its nonlinear component underestimated (Fig. 2, Suppl. Figs. S1, S2), potentially leading to some underestimation of the effect of atmospheric nonlinearities in the present study. Repeating a similar type of approach with different models would strengthen the robustness of the results.

Our results also suggest that oceanic nonlinearities act as a weak negative damping during extreme El Niño events, and therefore seem to contradict studies that have emphasized the role of oceanic nonlinearities in contributing to either ENSO asymmetry or extreme El Niño events^14,31–34^. It is indeed surprising that asymmetrical heating associated with phenomena like TIWs and NDH, as identified in re-analyses^31,33^, does not yield a more substantial contribution to ENSO asymmetry. We have thus used a similar strategy to the one reported here to explicitly quantify the effect of oceanic nonlinearities in the eastern Pacific. We will report those results elsewhere, but they also indicate a weak role of oceanic nonlinearities, which can be explained by the compensation between lateral and vertical processes.

However oceanic nonlinearities may be significant in the western Pacific. Our results indeed provide an explanation for the larger El Niño amplitude in the eastern Pacific, but not for the larger western Pacific La Niña amplitude. The negatively skewed western Pacific SST distribution is preserved when the ocean is forced by the linear part of wind stress anomalies. This strongly implies that oceanic nonlinearities play a pivotal role in shaping ENSO asymmetries in the western Pacific, warranting further investigation.

A final perspective of the current study can be drawn from a closer analysis of Fig. 5d. During the summer and fall that followed the extreme El Niño events, the initial development of the eastern Pacific negative SST anomaly, which later amplifies into a La Niña, is due to the response to nonlinear wind-stress anomalies. This may be due to the enhanced western Pacific discharge in response to the boreal fall and winter westerly wind-stress anomalies nonlinear component (Fig. 5b). This suggests a contribution of atmospheric nonlinearities to the observed more efficient discharge and more systematic phase transition to La Niña after extreme El Niño events^47^, a topic we will investigate in a future study.

## Methods

### Datasets and definition of anomalies

We use the following datasets for model validation: global precipitation climatology project (GPCP) v2.3 dataset^48^ for rainfall, Tropflux^49,50^ for surface momentum and heat fluxes, European Centre for Medium-Range Weather Forecasts (ECMWF) Ocean reanalysis system 5 (ORAS5) sea surface height^51^, and the Hadley Centre Global Sea Ice and Sea Surface Temperature (HadISST)^52^ SST. For these datasets and all simulations, we compute monthly anomalies by subtracting the 1980–2020 climatology and linearly de-trending.

The ENSO asymmetry is obtained as the half sum of extreme El Niño and strong La Niña composites (extreme El Niño: 1982–1983, 1997–1998 and 2015–2016; strong La Niña: 1988–1989, 1999–2000, 2000–2001, 2007–2008 and 2010–2011). Using all ENSO events yields very similar Figs. 2 and 3b–e, with a weaker amplitude (not shown).

### Atmospheric general circulation model control simulation

We use the atmospheric component of the Centre National de Recherches Météorologiques (CNRM) CNRM-CM6 climate model, which participated in the CMIP6 exercise^36^. ARPEGE-climate 6.3 is a global Atmospheric General Circulation Model (AGCM) spectral model with a T127 linear triangular truncation, equivalent to a spatial resolution of about 150 km, sufficient to capture the large-scale processes of relevance here. This model offers an increased vertical resolution (91 vertical levels and the top level at 0.01 hPa) and improved physical parameterizations (in particular moist processes involved in convection, as well as the turbulence and microphysics) relative to its previous release^53^. In particular, the new Prognostic Condensates, Microphysics and Transport (PCMT) convection scheme^54,55^, provides a consistent, continuous and prognostic treatment of convection from dry thermals to deep precipitating events, smoothing the transition between shallow and deep convection.

Our control atmospheric experiment spans the 1979 to 2020 period and is forced by observed HadISST^52^ data and the observed evolution of the atmospheric chemical composition, including greenhouse gases. We performed a 6-member ensemble simulation. Based on a Monte Carlo approach, we estimated that the ensemble average monthly Niño3 and Niño4 wind-stress anomalies are within 20% of the converged value that could be achieved with a larger ensemble.

### Estimating the linear and nonlinear components of the atmospheric response to SST

We conducted an identical 6-member ensemble AGCM experiment to the one described above, but with reversed-sign SST anomalies sign (i.e. we forced the model by the SST climatology $\overline{T }$ *minus* the SST anomalies $T^{\prime}$). The reversed SST anomalies were ramped down to zero poleward of 60° latitudes, to avoid reaching SST below the freezing point of seawater. If the AGCM response to SST was purely linear, the anomalous atmospheric response $A\left(-T^{\prime}\right)$ of the experiment forced by $\overline{T }-T^{\prime}$ would be opposite to that forced by $\overline{T }+T^{\prime}$, but this is not the case (see the Suppl. Fig. S6 example). In principle $1/2(A\left({T}^{\prime}\right)-A\left(-{T}^{\prime}\right))$ gives the sum of all the odd terms in the Taylor expansion of $A\left({T}^{\prime}\right)$ and $1/2(A\left({T}^{\prime}\right)+A\left(-{T}^{\prime}\right))$ the even terms. In practice (see main text and Fig. 1), the former is dominated by the 1^st^ order linear term, and the second by the second-order quadratic term. We hence simply refer to them as the linear and nonlinear atmospheric response to SST anomalies, respectively.

### Oceanic model control simulation

We use the NEMO v3.6 (Nucleus for European Models of the Ocean)^46^ Ocean General Circulation Model (OGCM), which is the oceanic component of the CNRM–CM6.1 climate model^36^. This model version features a 1° horizontal resolution, with a latitudinal grid refinement to 1/3° within the equatorial band. The ocean vertical mixing of tracers and momentum is parameterized by a turbulent kinetic energy scheme^56^, and oceanic convection is represented by a strong increase of the tracer’s vertical diffusion coefficient when static instability is present.

We perform a single control simulation with the NEMOv3.6 oceanic model driven by interannual-varying momentum and fresh water air-sea fluxes from the ocean forcing product derived from the Japanese 55-year reanalysis (JRA55-do)^57^ over the 1979–2020 period. In the equatorial Pacific, net air-sea fluxes Q can closely be approximated by $\overline{Q}-\gamma \left(T-\overline{T }\right)$ where the first term is the JRA55-do net heat flux climatology and the second relaxation of the model SST $T$ to a climatological observed SST $\overline{T }$ with a − 15 Wm^−2^ C^−1^ coefficient^45^. The surface heat fluxes are interactively computed by the ocean model, using this approach. The model has thus a relaxation to the observed climatological SST, but not to its interannual anomalies, allowing an independent evaluation of the model SST to observations. This approach produces SST anomalies that are very close to the observed values in the Niño3 (and Niño4, not shown) region (Fig. 3a, the model just slightly overestimates the peak warming during extreme El Niño events).

### Oceanic response to the linear and nonlinear wind stresses; mixed layer budget

Apart from the control experiment mentioned above, we also conducted OGCM sensitivity experiments aimed at quantifying the ocean response to the linear and nonlinear parts of the wind-stress anomalies. The “linear τ(SST)” experiment is obtained by subtracting the nonlinear wind stress response to SST diagnosed from the AGCM experiments ${\overrightarrow{\tau }}_{nl}^{\prime}$ from the JRA55-do forcing. It is otherwise in all points similar to the control experiment. The oceanic response to the atmospheric nonlinearities is obtained through the “nonlinear τ(SST)” experiment. This experiment is similar to the control experiment, but the wind-stress forcing is this time obtained by adding ${\overrightarrow{\tau }}_{nl}^{\prime}$ to the JRA55-do 1980–2020 climatological wind-stress. The sum of the wind stress anomalies from these two experiments by construction adds up to the JRA-do wind stress anomalies used to force the control OGCM experiment.

To analyse the processes that control SST in those experiments, we performed a mixed heat budget analysis as in Vialard et al.^45^. The tendency terms computed at each time step by the ocean model are vertically integrated over the time-varying mixed layer depth, defined as the depth with a 0.01 kg m^−3^ density increase relative to the density at 10 m. Results are robust when using other reasonable definitions of the mixed layer (not shown). The same procedure as that in Vialard et al.^45^ allows decomposing the time derivative of SST anomalies into contributions from (a) low-frequency lateral advection, (b) air-sea fluxes, (c) subsurface processes (that gather upwelling and vertical mixing through the mixed layer bottom and entrainment) and (d) Tropical Instability Waves (TIWs). TIWs contributions group the contribution of time scales shorter than one month to lateral advection and a weak lateral mixing term from the model parameterization of subgrid-scale processes. Low-frequency advection lateral advection includes contributions from time scales longer than one month. The computation of this budget during the model integration allows an exactly closed budget. Interannual anomalies of this budget in the second experiment described above are depicted in Fig. 5, providing insights into how wind-stress nonlinearities contribute to SST changes.

### Supplementary Information

Supplementary Information.

## Data Availability

The numerical simulations from the atmospheric and ocean models used in this manuscript are available on request from the corresponding author.

## References

[1] Vincent EM (2011). Interannual variability of the South Pacific Convergence Zone and implications for tropical cyclone genesis. Clim. Dyn..

[2] Cai W, Lengaigne M, Borlace S, Collins M, Cowan T, McPhaden MJ (2012). More extreme swings of the South Pacific convergence zone due to greenhouse warming. Nature.

[3] Beniche M, Vialard J, Lengaigne M, Voldoire A, Srinivas G, Hall N (2023). A distinct and reproducible teleconnection pattern over North America during extreme El Niño events. Sci. Rep..

[4] Jong B-T, Ting M, Seager RE (2016). Niño’s impact on California precipitation: Seasonality, regionality, and El Niño intensity. Environ. Res. Lett..

[5] Hoell A (2016). Does El Niño intensity matter for California precipitation?. Geophys. Res. Lett..

[6] Lee S-K, Lopez H, Chung E-S, DiNezio P, Yeh S-W, Wittenberg AT (2018). On the fragile relationship between El Niño and California rainfall. Geophys. Res. Lett..

[7] Santoso A, Mcphaden MJ, Cai W (2017). The defining characteristics of ENSO extremes and the strong 2015/2016 El Niño. Rev. Geophys..

[8] Bayr T, Lübbecke JF, Vialard J, Latif M (2024). Equatorial pacific cold tongue bias degrades the simulation of ENSO asymmetry in climate models. J. Clim..

[9] An SI, Tziperman E, Okumura YM, Li T, McPhaden MJ, Santoso A, Cai W (2020). ENSO irregularity and asymmetry. El Niño Southern Oscillation in a Changing Climate.

[10] Okumura YM, Deser C (2010). Asymmetry in the duration of El Niño and La Niña. J. Clim..

[11] Li X, Hu ZZ, McPhaden MJ, Zhu C, Liu Y (2023). Triple-Dip La Niñas in 1998–2001 and 2020–2023: Impact of mean state changes. J. Geophys. Res. Atmos..

[12] Capotondi A, Wittenberg AT, Kug JS, Takahashi K, McPhaden MJ, McPhaden MJ, Santoso A, Cai W (2021). ENSO diversity. El Niño Southern Oscillation in a Changing Climate.

[13] Geng T, Cai W, Wu L, Yang Y (2019). Atmospheric convection dominates genesis of ENSO asymmetry. Geophys. Res. Lett..

[14] Timmermann A, Jin FF, Abshagen J (2003). A nonlinear theory for El Niño bursting. J. Atmos. Sci..

[15] Takahashi K, Karamperidou C, Dewitte B (2019). A theoretical model of strong and moderate El Niño regimes. Clim. Dyn..

[16] Gadgil S, Joseph PV, Joshi NV (1984). Ocean–atmosphere coupling over monsoon regions. Nature.

[17] Graham NE, Barnett TP (1987). Surface temperature, surface wind divergence, and convection over tropical oceans. Science.

[18] Hoerling MP, Kumar A, Zhong M (1997). El Niño, La Niña, and the nonlinearity of their teleconnections. J. Clim..

[19] Srinivas G, Vialard J, Lengaigne M, Izumo T, Guilyardi E (2022). Relative contributions of sea surface temperature and atmospheric nonlinearities to ENSO asymmetrical rainfall response. J. Clim..

[20] Kang I-S, Kug J-S (2002). El Niño and La Niña sea surface temperature anomalies: Asymmetry characteristics associated with their wind stress anomalies. J. Geophys. Res..

[21] Frauen C, Dommenget D (2010). El Niño and la Niña amplitude asymmetry caused by atmospheric feedbacks. Geophys. Res. Lett.

[22] Choi KY, Vecchi GA, Wittenberg AT (2013). ENSO transition, duration, and amplitude asymmetries: Role of the nonlinear wind stress coupling in a conceptual model. J. Clim..

[23] Lopez H, Kirtman BP (2015). Tropical pacific internal atmospheric dynamics and resolution in a coupled GCM. Clim. Dyn..

[24] Takahashi K, Dewitte B (2016). Strong and moderate nonlinear El Niño regimes. Clim. Dyn..

[25] Harrison DE, Vecchi GA (1999). On the termination of El Niño. Geophys. Res. Lett..

[26] Eisenman I, Yu LS, Tziperman E (2005). Westerly wind bursts: ENSO’s tail rather than the dog?. J. Clim..

[27] Puy M, Vialard J, Lengaigne M, Guilyardi E (2016). Modulation of equatorial Pacific westerly/easterly wind events by the Madden–Julian oscillation and convectively-coupled Rossby waves. Clim. Dyn..

[28] Lopez H, Kirtman BP (2013). Westerly wind bursts and the diversity of ENSO in CCSM3 and CCSM4. Geophys. Res. Lett..

[29] Jin F-F, Lin L, Timmermann A, Zhao J (2007). Ensemble-mean dynamics of the ENSO recharge oscillator under state-dependent stochastic forcing. Geophys. Res. Lett..

[30] Zebiak SE, Cane MA (1987). A model El Niño-Southern oscillation. Mon. Weather Rev..

[31] An S-I (2008). Interannual variations of the tropical ocean instability wave & ENSO. J. Clim..

[32] Xue A, Zhang W, Boucharel J, Jin FF (2021). Anomalous tropical instability wave activity hindered the development of the 2016/17 La Niña. J. Clim..

[33] An S-I, Jin F-F (2004). Nonlinearity and asymmetry of ENSO. J. Clim..

[34] An S-I, Kim J-W (2017). Role of nonlinear ocean dynamic response to wind on the asymmetrical transition of El Niño and La Niña. Geophys. Res. Lett..

[35] Timmermann A, Jin F-F (2002). Phytoplankton influences on tropical climate. Geophys. Res. Lett..

[36] Voldoire A (2019). Evaluation of CMIP6 DECK experiments with CNRM-CM6-1. J. Adv. Model. Earth Syst..

[37] Jin FF, Chen HC, Zhao S, Hayashi M, Karamperidou C, Stuecker MF, McPhaden MJ, Santoso A, Cai W (2020). Simple ENSO models. El Niño Southern Oscillation in a Changing Climate.

[38] Zebiak SE (1986). Atmospheric convergence feedback in a simple model for El Niño. Mon. Weather Rev..

[39] Vecchi GA, Harrison DE (2006). The termination of the 1997–98 El Niño. Part I: Mechanisms of oceanic change. J. Clim..

[40] Vecchi GA (2006). The termination of the 1997–98 El Niño. Part II: Mechanisms of atmospheric change. J. Clim..

[41] Lengaigne M, Vecchi GA (2010). Contrasting the termination of moderate and extreme El Niño events in coupled general circulation models. Clim. Dyn..

[42] Jin FF (1997). An equatorial ocean recharge paradigm for ENSO. Part I: Conceptual model. J. Atmos. Sci..

[43] Jin FF (1997). An equatorial ocean recharge paradigm for ENSO. Part II: A stripped-down coupled model. J. Atmos. Sci..

[44] Weisberg RH, Wang C (1997). Slow variability in the equatorial west-central Pacific in relation to ENSO. J. Clim..

[45] Vialard J, Menkes C, Boulanger JP, Delecluse P, Guilyardi E, McPhaden MJ, Madec G (2001). A model study of oceanic mechanisms affecting equatorial Pacific sea surface temperature during the 1997–98 El Niño. J. Phys. Oceanogr..

[46] Madec G (2017). Zenodo..

[47] Iwakiri T, Watanabe M (2021). Mechanisms linking multi-year La Niña with preceding strong El Niño. Sci. Rep..

[48] Adler R (2018). The global precipitation climatology project (GPCP) monthly analysis (new version 2.3) and a review of 2017 global precipitation. Atmosphere.

[49] Praveen Kumar B, Vialard J, Lengaigne M, Murty VSN, Mcphaden MJ, Cronin MF (2013). TropFlux wind stresses over the tropical oceans: Evaluation and comparison with other products. Clim. Dyn..

[50] Praveen Kumar B, Vialard J, Lengaigne M, Murty VSN, Mcphaden MJ (2012). TropFlux: Air-sea fluxes for the global tropical oceans—Description and evaluation. Clim. Dyn..

[51] Zuo H, Balmaseda MA, Tietsche S, Mogensen K, Mayer M (2019). The ECMWF operational ensemble reanalysis–analysis system for ocean and sea ice: A description of the system and assessment. Ocean Sci..

[52] Rayner NA, Parker DE, Horton EB, Folland CK, Alexander LV, Rowell DP, Kent EC, Kaplan A (2003). Global analyses of sea surface temperature, sea ice, and night marine air temperature since the late nineteenth century. J. Geophys. Res. Atmos..

[53] Roehrig R, Beau I, Saint-Martin D, Alias A, Decharme B, Guérémy JF (2020). The CNRM global atmosphere model ARPEGE-Climat 6.3: Description and evaluation. J. Adv. Model. Earth Syst..

[54] Piriou J-M, Redelsperger J-L, Geleyn J-F, Lafore J-P, Guichard F (2007). An approach for convective parameterization with memory: Separating microphysics and transport in grid-scale equations. J. Atmos. Sci..

[55] Guérémy J-F (2011). A continuous buoyancy based convection scheme: One- and three-dimensional validation. Tellus A.

[56] Blanke B, Delecluse P (1993). Low-frequency variability of the tropical Atlantic ocean simulated by a general circulation model with mixed layer physics. J. Phys. Oceanogr..

[57] Tsujino H, Urakawa S, Nakano H, Small RJ, Kim WM, Yeager SG (2018). JRA-55 based surface dataset for driving ocean–sea-ice models (JRA55-do). Ocean Model..

