# Supplementary material for: Dominant contribution of atmospheric nonlinearities to ENSO asymmetry and extreme El Niño events
Source: Sci Rep. 2024 Apr 7;14:8122. doi: 10.1038/s41598-024-58803-3 (PMC10998846; doi:10.1038/s41598-024-58803-3)
Supplement: Supplementary file 1 — Supplementary Information. [file 41598_2024_58803_MOESM1_ESM.docx]

**Supplementary Information**

| **NDJ (FMA)** | **Linear τ(SST) %** | **Nonlinear τ(SST) %** | **Oceanic NL %** |
| --- | --- | --- | --- |
| **1982-83** | 81 (65) | 44 (50) | -25 (-15) |
| **1997-98** | 78 (77) | 44 (45) | -22 (-23) |
| **2015-16** | 72 (37) | 36 (72) | -8 (-8) |
| **composites** | 77 (59) | 41 (56) | -18 (-15) |

**Table S1**. Percentage of contributions of linear τ(SST), nonlinear τ(SST) and oceanic nonlinearities to the November through January (NDJ) average Niño3 SST anomalies, for the 1982-83, 1997-98 and 2015-16 extreme El Niño events and their composite mean. The contributions are respectively estimated from Figure 4 as linear τ(SST), nonlinear τ(SST), and CTL minus (linear τ(SST) + nonlinear τ(SST)) divided by the CTL experiment. The number in brackets provides the same quantities for the February through April average (FMA). Part of the information in Table S1 is indicated above each extreme El Niño in Figure 4 and corresponds to numbers quoted in the text and abstract. Note that Figure 5e gives a larger contribution from atmospheric nonlinearities, because *i)* it gives contributions to SST anomalies *changes* over the last 6 months of the year and *ii)* it was tailored to analyse the physical processes associated with the response to the nonlinear component of the wind stress over regions and periods for which this contribution is largest. The numbers in Figure 4 and Table S1 are better estimates of the overall contribution of atmospheric nonlinearities to extreme El Niño peak anomalies.

***Key message****. Overall, the wind stress nonlinearities contribute to about 40% of the NDJ and ~55% of the FMA SST anomalies during the three extreme El Niño events. Oceanic nonlinearities tend to dampen the total SSTA by about 15%.*


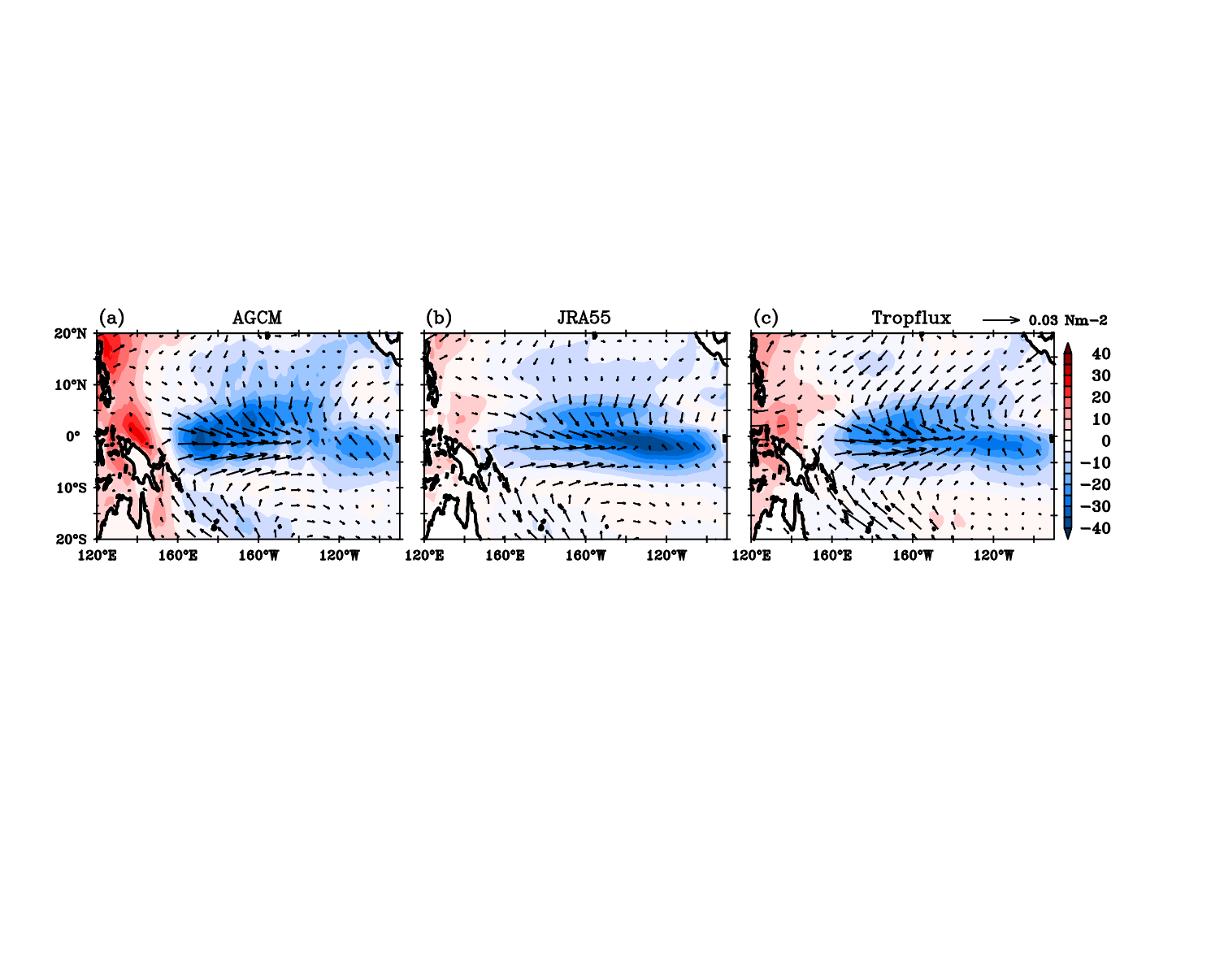


**Figure S1.** Regression of net heat flux (shaded, w/m^2^) and wind stress (vectors, N/m^2^) anomalies on Nino3.4 SST anomalies from **(a)** control AGCM ensemble mean, **(b)** JRA55-do and **(c)** TropFlux.

***Key message****. The typical ENSO total wind stress anomalies in our ensemble AGCM experiment (used to estimate the nonlinear wind stress response to SST) are very similar to that used to force the CTL OGCM experiment (JRA55-do) and that of another product (Tropflux). The wind stress – SST feedback (estimated as the average Niño4 wind stress on Niño3.4) for the AGCM ensemble is indeed 0.016 Nm^-2^°C^-1^ against 0.013 / 0.016 for JRA55-do and Tropflux, respectively. This figure also justifies the -15 Wm^-2^K^-1^ feedback coefficient we use to compute the interannual net heat flux anomalies associated with ENSO in our experiments (note, however, that the value of this coefficient may be too low in the central and western Pacific, which may explain why we tend to overestimate SST anomalies during some events).*


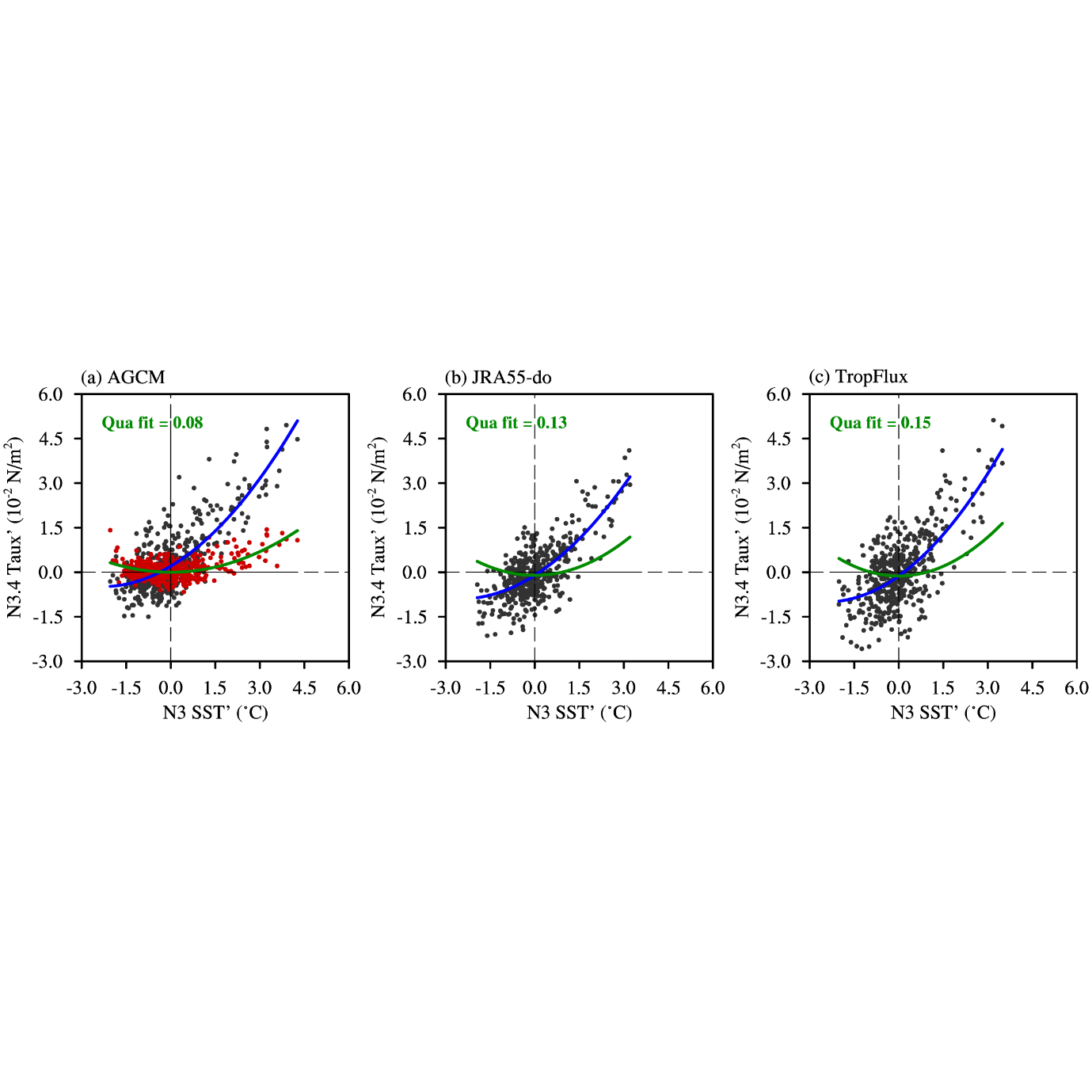


**Figure S2**. Scatter diagram of Niño3+4 region (160°E-90°W, 5°N-5°S) zonal wind stress anomalies (black dots) against average Niño3 (150°W-90°W, 5°N-5°S) SST anomalies from **(a)** AGCM, **(b)** JRA55-do and **(c)** TropFlux. The red dots on panel (a) show the atmospheric nonlinear contributions to the wind stress response to SST anomalies estimated from the ensemble AGCM experiments as detailed in the Methods. The blue curve is a quadratic fit (a+b T + c T^2^) of the wind stress response to SST anomalies, and the green curve the isolated quadratic component (c T^2^) with the value of c indicated in green on each panel. The Niño3+4 region was chosen for the y-axis because it captures wind stress variations during both El Niño and La Niña. The NiNno3 regions was chosen for the x-axis because that is the region where large SST anomalies are requested to excess the deep atmospheric convection threshold.

***Key message****. The quadratic component of the average Niño3+4 wind stress anomalous response to SST anomalies deducted from our AGCM experiments is quite consistent with values estimated from observations (JRA55-do and Tropflux). If anything, the amplitude of the nonlinear wind stress response to SST is underestimated, suggesting that its contribution to extreme El Niño events and the overall ENSO asymmetry may be a bit larger in reality than in estimates from the current paper.*


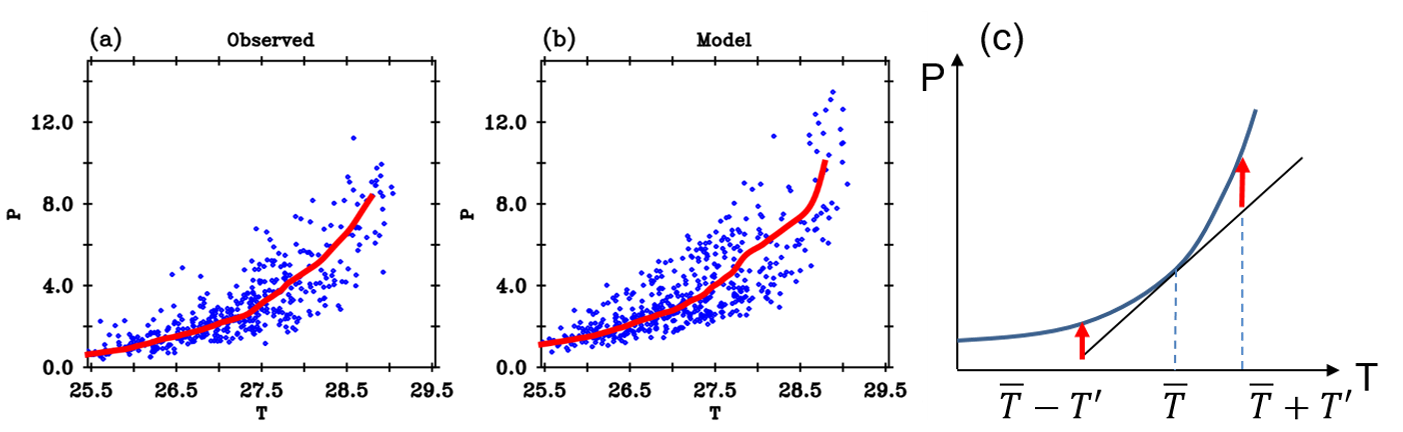


**Figure S3.** The rainfall – SST (P(T)) relationship over the Niño3+4 (160°E-90°W, 5°N-5°S) region in **(a)** observations and **(b)** our AGCM ensemble simulation. The schematic representation in **(c)** explains why nonlinearities tend to be positive for both positive and negative SST anomalies (T’). The red curve on panels (a) and (b) shows the median of the nonlinear P(T) relationship.

***Key message****. The median red curve on panels a and b indicates that rainfall increases more or less exponentially with climatological SST in observations and our AGCM ensemble experiment (also see Srinivas et al. (2022)). This P(T) relation has a convex shape. Panel (c) shows that the P(T) relation always lies above the tangent curve, i.e. those nonlinearities (red arrows, corresponding to deviations from the linear tangent curve) are positive for both positive and negative SST anomalies T’, i.e. are dominated by quadratic nonlinearities. Srinivas et al. (2022) performed a vertically integrated moisture budget in an atmospheric re-analysis and showed that those nonlinearities correspond to both the convergence feedback (the fact that the rainfall anomaly enhances the low-level convergence) and to the anomalous convergence of anomalous moisture terms.*


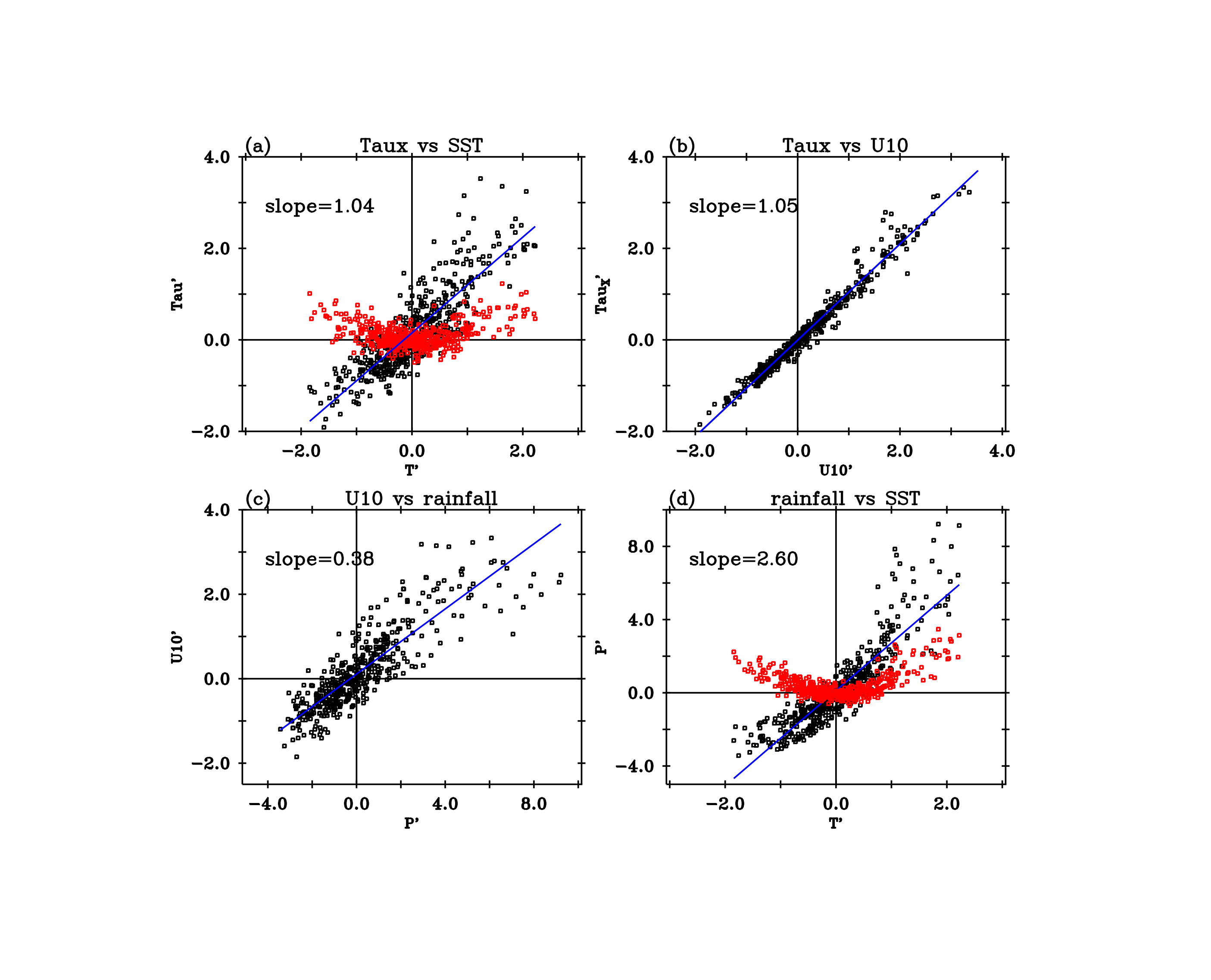


**Figure S4**. Scatterplot of Niño3+4 (160°E- 90°W, 5°N-5°S) average (black dots) **(a)** zonal wind stress (10^-2^ Nm^-2^) vs SST (°C), **(b)** zonal wind stress vs surface zonal wind (m/s), **(c)** surface zonal wind vs rainfall (mm/day), and **(d)** rainfall vs SST. The blue line on each panel is a linear fit, and the red dots on **a** and **d** indicate the nonlinear part of the response to SST.

***Key message****. Panel a shows the anomalous zonal wind stress-SST relation of Figure 2b. The nonlinearity in this relation can either arise from the bulk formula that links wind stress to surface wind (panel b); from potential nonlinearities of the surface wind response to rainfall, a measure of vertically integrated tropospheric heating, (panel c); or from the relation between rainfall and SST (panel d, similar to Fig. 2a). The zonal wind stress anomalies scale very linearly with the zonal surface wind anomalies (b) as well as surface wind with rainfall (c). Most of the nonlinearity in the wind stress – SST relation hence arises from that in the rainfall-SST anomalies relation.*


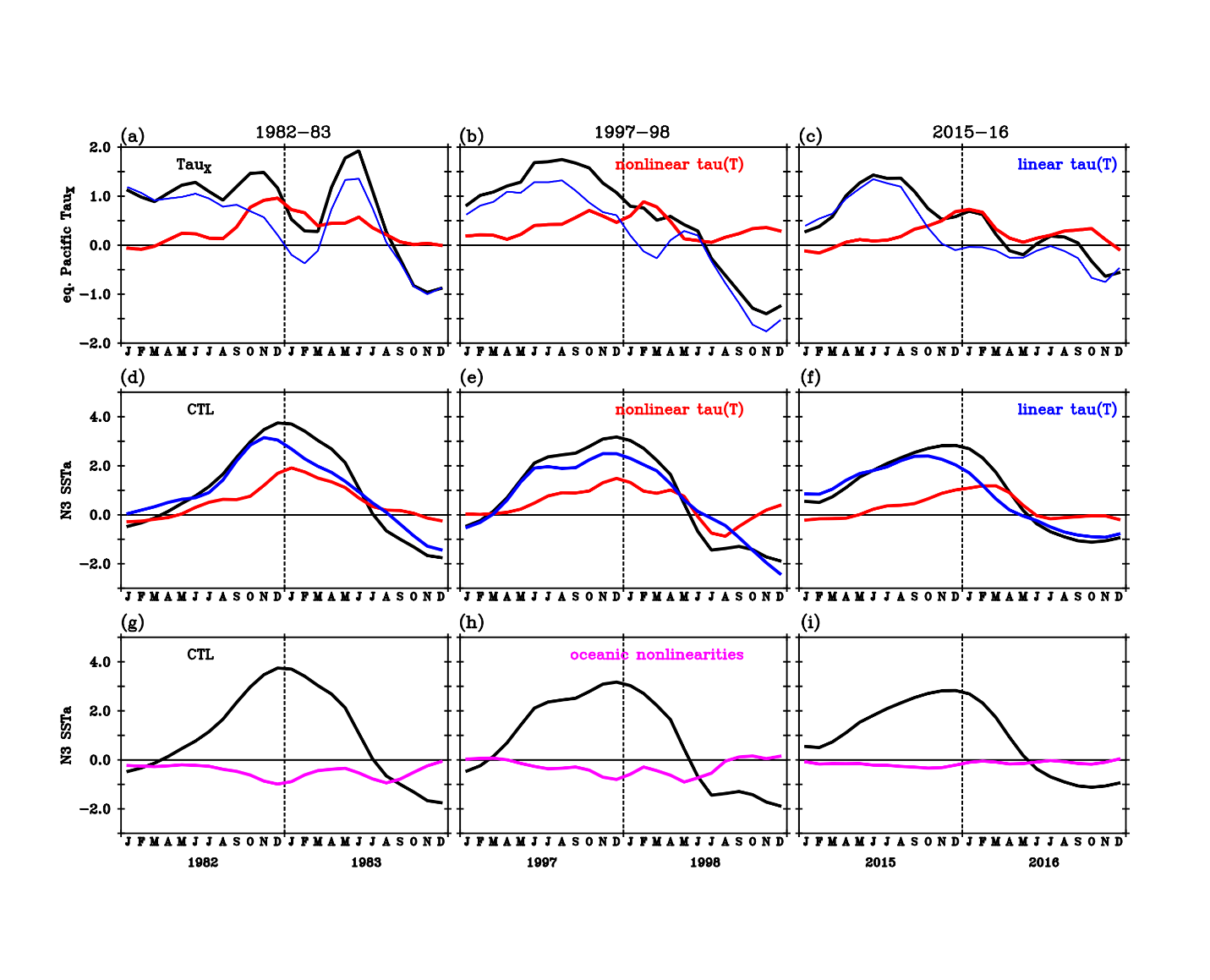


**Figure S5.** The interannual time series of equatorial Pacific (5°N-5°S, 120°E-80°W) average zonal wind stress anomalies (10^-2^ Nm^-2^) with linear (blue) and nonlinear (red) components of the response to SST during the **(a)** 1982-83, **(b)** 1997-98 and **(c)** 2015-16 extreme El Nino events. **(d)- (f)** same as (a) - (c) but for Niño3 SST anomalies (°C) from the control OGCM simulation (black), and contributions from the linear (blue, linear τ (SST) experiment) and nonlinear (red, nonlinear τ (SST) experiment) wind stress response to SST. **(g)-(i)** same as (a) – (c) but for Nino3 SST anomalies from the control OGCM (black) and oceanic nonlinearities (magenta, obtained as the control minus the sum of the linear and nonlinear τ (SST) experiments).

***Key message****. This figure is a zoomed version of the main manuscript figure 4 for the 3 extreme El Niño in the time series. It allows us to appreciate the large wind stress nonlinearities contribution to the extreme El Niño peak and prolonged warming, which is further quantified by Table S1 (about 40 and 55%, respectively).*


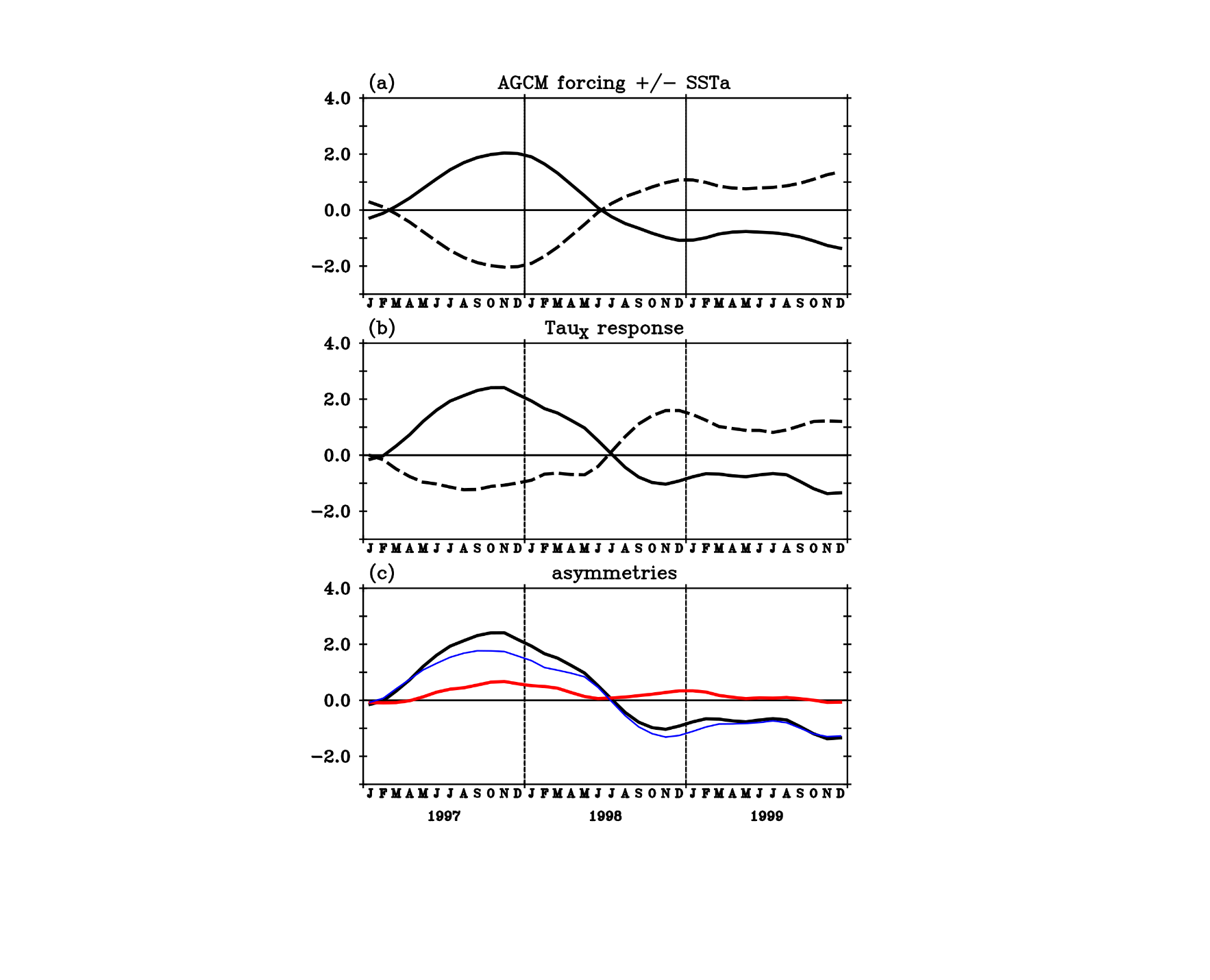


**Figure S6. Atmospheric anomalies decomposition into linear and nonlinear response to SST.** Average Niño3+4 (160°E to 90°W, 5°N to 5°S) interannual anomalies during 1997-1999: **(a)** SST (continuous) and its opposite (dashed), used to force the two 6-member ensemble AGCM experiments. **(b)** Zonal wind stress response to observed SST anomalies (black) and their opposite (dashed). Notice the smaller response to cold SST anomalies. **(c)** total wind stress anomalies (black) and linear (blue) and nonlinear (red) components of the response to SST.

***Key message****. This illustrates our method for decomposing the atmospheric response to SST (black curve on panel c) into a linear (blue) and nonlinear (red) part. In principle, the red curve contains the odd terms in the Taylor expansion, but the 1^st^ order term dominates. The blue curve is similarly dominated by the quadratic term (first even term in the Taylor expansion, see Fig 1bd). With only 6 members, there is some aliasing of the low-frequency response by internal atmospheric variability, and a better estimate would be obtained with more members.*
